# An empirical comparison of population genetic analyses using microsatellite and SNP data for a species of conservation concern

**DOI:** 10.1186/s12864-020-06783-9

**Published:** 2020-06-01

**Authors:** Shawna J. Zimmerman, Cameron L. Aldridge, Sara J. Oyler-McCance

**Affiliations:** 1 U.S. Geological Survey, Fort Collins Science Center, 2150 Centre Avenue, Bldg. C, Fort Collins, CO 80526 USA; 2 Department of Ecosystem Science and Sustainability and Natural Resource Ecology Laboratory, Colorado State University, Fort Collins, CO 80526 USA

**Keywords:** Population structure, Population genetics, Evolutionarily significant units, Conservation, Genomics, Microsatellites, Single nucleotide polymorphisms

## Abstract

**Background:**

Use of genomic tools to characterize wildlife populations has increased in recent years. In the past, genetic characterization has been accomplished with more traditional genetic tools (e.g., microsatellites). The explosion of genomic methods and the subsequent creation of large SNP datasets has led to the promise of increased precision in population genetic parameter estimates and identification of demographically and evolutionarily independent groups, as well as questions about the future usefulness of the more traditional genetic tools. At present, few empirical comparisons of population genetic parameters and clustering analyses performed with microsatellites and SNPs have been conducted.

**Results:**

Here we used microsatellite and SNP data generated from Gunnison sage-grouse (*Centrocercus minimus*) samples to evaluate concordance of the results obtained from each dataset for common metrics of genetic diversity (*H*_O_, *H*_E_, *F*_IS_, *A*_R_) and differentiation (*F*_ST_, *G*_ST_, *D*_Jost_). Additionally, we evaluated clustering of individuals using putatively neutral (SNPs and microsatellites), putatively adaptive, and a combined dataset of putatively neutral and adaptive loci. We took particular interest in the conservation implications of any differences. Generally, we found high concordance between microsatellites and SNPs for *H*_E_, *F*_IS_, *A*_R_, and all differentiation estimates. Although there was strong correlation between metrics from SNPs and microsatellites, the magnitude of the diversity and differentiation metrics were quite different in some cases. Clustering analyses also showed similar patterns, though SNP data was able to cluster individuals into more distinct groups. Importantly, clustering analyses with SNP data suggest strong demographic independence among the six distinct populations of Gunnison sage-grouse with some indication of evolutionary independence in two or three populations; a finding that was not revealed by microsatellite data.

**Conclusion:**

We demonstrate that SNPs have three main advantages over microsatellites: more precise estimates of population-level diversity, higher power to identify groups in clustering methods, and the ability to consider local adaptation. This study adds to a growing body of work comparing the use of SNPs and microsatellites to evaluate genetic diversity and differentiation for a species of conservation concern with relatively high population structure and using the most common method of obtaining SNP genotypes for non-model organisms.

## Background

Accurate estimation of population genetic parameters has become an important part of wildlife conservation [1]. Genetic characterization can be used to identify populations and understand gene flow [2–5]. More recently, genetic data have been used to begin to understand local adaptation [6–8] and to identify groups with distinct evolutionary or demographic characteristics [9–12]. Most past genetic studies of wildlife species have been accomplished with relatively few (< 20) highly variable microsatellite loci. Microsatellites, also called simple sequence repeats, were discovered in the 1980s and were quickly adopted as one of the most commonly used genetic markers [13, 14] because they tend to be highly polymorphic, are evenly distributed throughout the genome [15, 16], and are located in non-coding regions allowing the general assumption that neutral processes were being meausured. Unlike many other types of markers, microsatellites have a high mutation rate that is quite variable across different loci. This mutation rate is the result of slippage during DNA replication, a process that is not well understood [17]. The high mutation rate of microsatellites that results in highly informative markers may also lead to an underestimate of heterozygosity through homoplasy, or when two individuals have the same allelic state through independent mutation and not from a common ancestor [17]. Additionally, repeatability of genotyping across laboratories can be challenging [18–21] largely because allele size calls are somewhat subjective and size determination methods can impact inferred fragment size [22], even with use of automated software [23].

A single nucleotide polymorphism (SNP) is a location in the DNA sequence where individuals vary at a single nucleotide. Technological advancements have allowed creation of much larger SNP genotype datasets, greatly increasing the number of loci sampled with less effort and lower cost in comparison to microsatellite development and genotyping [16]. Because of their high prevalence in the genome and the potential to target functional regions, SNPs are predicted to replace microsatellites for genetic characterization [24]. SNPs are more abundant and uniformly distributed across the genome than microsatellites, and have a well-understood mutational mechanism with low levels of homoplasy [25], but they have lower allelic diversity [26]. Lower allelic diversity in comparison to microsatellites is expected, because a nucleotide base at a SNP can only be one of four possible states: A, T, C, or G. In reality, the natural pairing of certain bases in DNA structure and the low likelihood of multiple mutations at one location results in the majority of SNPs being biallelic. Because of the relatively low allelic diversity, equal distribution throughout the genome, ascertainment bias of highly polymorphic microsatellite regions, and relatively constant mutation rate of SNPs, some have argued that SNPs provide a more accurate representation of genome-wide variation [27, 28]. Until recently, SNP datasets were only available for species with reference genomes, such as model organisms or important agricultural species. The development of reduced representation methods to obtain SNP genotypes without a reference genome has broadened the application of SNP markers to numerous species [29, 30]. One of the main appeals of SNP loci is the ease with which high throughput/automatic analyses can be used in comparison with development and genotyping of microsatellites [24, 31, 32] resulting in the generation of large numbers of genotypes in a relatively short period of time and for minimal cost. Further, increasing the number of loci sampled is expected to increase precision of population genetic estimates [33, 34].

In addition to the potential improvement in precision of population parameter estimates from the increased number of loci, the explosion of genomic techniques and their application to non-model organisms has also led to the ability to ask new questions about conservation [35, 36]. SNPs are found in coding and non-coding regions of the genome and they can represent both demographic (i.e., drift) and functional (i.e., selection) processes. Many authors have suggested that conservation units identified below the species level should incorporate an evaluation of demographic and evolutionary distinctness [37–41]. Defining genetically similar units for conservation can inform management actions (e.g., habitat restoration, translocation) or potentially impact legal protection status under the Endangered Species Act (ESA), which allows for the separate protection of geographically and ecologically distinct populations [42]. The predicted advantages to using SNP data as opposed to microsatellite data for conservation have lead us to question if microsatellites will be a useful tool in the future or will be completely replaced by SNP data.

Technological advancements in genomic approaches for non-model organisms has resulted in use of reduced representation sequencing methods to generate large SNP datasets for many wildlife species; datasets that are often archived and available for potential future use. Understanding how SNP data compare to inferences made from the more traditional microsatellite data is important for long-term genetic monitoring given the increasing trend of using SNP data for conservation objectives. Previous studies have compared the relative abilities of SNP and microsatellite loci to evaluate levels of relatedness [43–49], probability of identity and parentage [50–54], create linkage maps [55, 56], evaluate genetic diversity [43, 45, 46, 51, 57–61], and detect low to mid levels of differentiation [45, 57–62]. Some studies have even used genome-wide SNP data to identify distinct population units [11, 12, 63, 64]. Here we used SNP and microsatellite datasets from the same group of genetic samples from a species of conservation concern to empirically evaluate agreement across marker types for population genetic analyses and consider the potential consequences in conservation decision making. The samples we used are typical of many conservation studies: opportunistically collected, variable source, variable quality, and from multiple populations of variable size that are represented by variable numbers of samples. Additionally, we used previously identified candidate adaptive loci [65] to evaluate identification of distinct units using datasets composed of genetic markers reflecting different evolutionary processes.

**Fig. 1:**
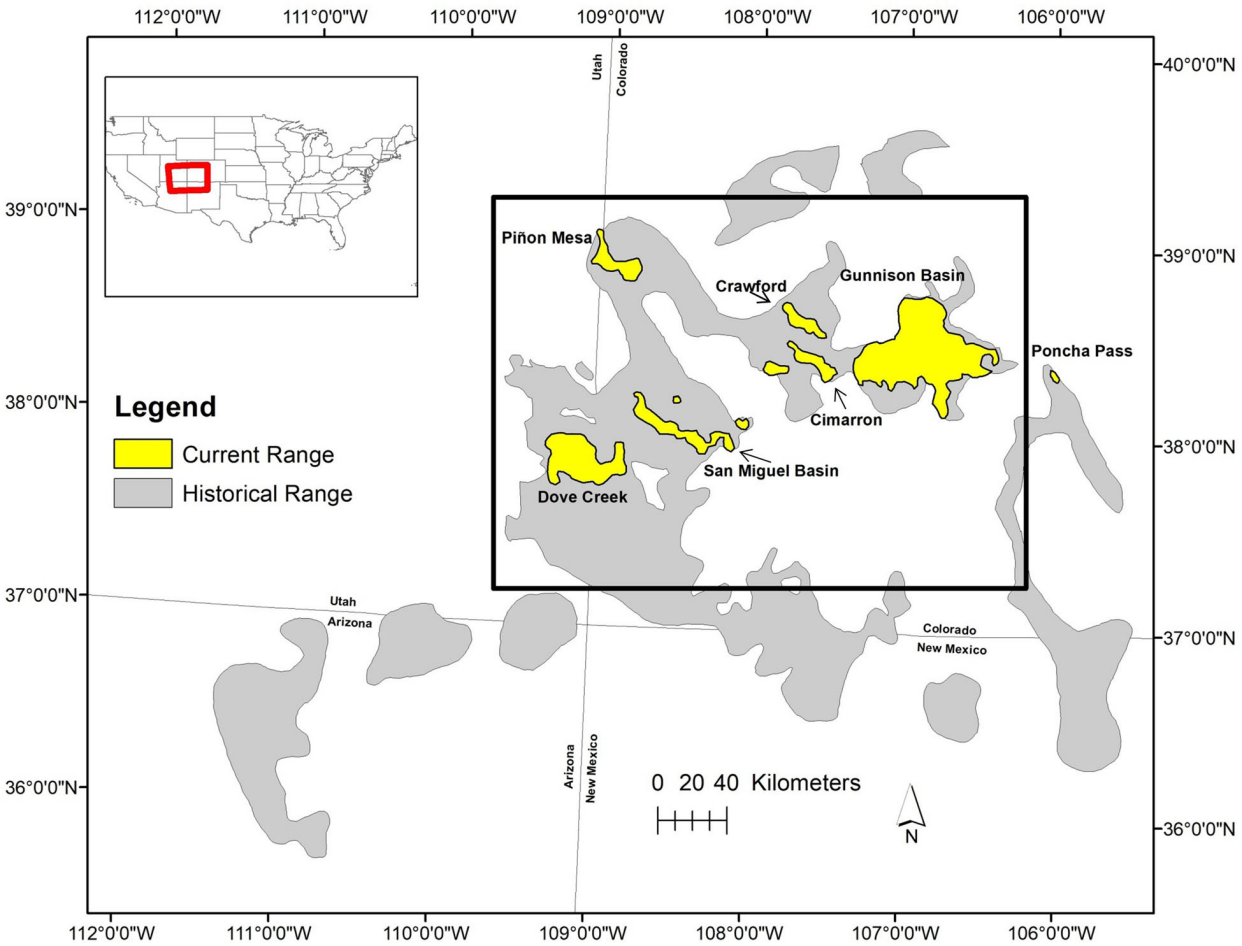
Gunnison sage-grouse distribution. Historical (gray) and current (yellow) distribution of Gunnison sage-grouse in the southwestern United States. Populations are labeled with respective names. Black rectangle designates the study area. Spatial data files were originally developed by Schroeder et al. [66], and the present map created by SJZ in ArcMap 10.1. The historical range map is as described by Braun et al. [67]; the two northernmost portions of the historical range correspond to an unknown species of sage-grouse and are not verified by Colorado Parks and Wildlife [68]

The Gunnison sage-grouse (*Centrocercus minimus*) is a sagebrush obligate avian species listed as threatened under the Endangered Species Act in 2014. The species exists as a network of seven populations predominantly occurring in Colorado and a small portion of the range extending into Utah (Fig. 1) [66, 67]. The majority of individuals in the species (~ 85–90%) are located in the Gunnison Basin population, which is largest in land area and highest in genetic diversity [69]. The six remaining satellite populations support much smaller numbers of birds; in descending order San Miguel Basin, Piñon Mesa, Crawford, Dove Creek-Monticello (Dove Creek from here on), Cerro Summit-Cimarron-Sims Mesa (Cimarron from here on), and Poncha Pass (Table 1) [70]. Genetic differentiation is high between all populations [69], local environmental conditions are variable [68], and there is some evidence of adaptive divergence among populations [65]. The Poncha Pass population is thought to have been extirpated in the 1970s, re-established with individuals translocated from Gunnison Basin, and currently persists as the result of on-going translocations [71]. Consequently, the Poncha Pass population was not included in the analyses presented here.

**Table 1:** Sample size for each population of Gunnison sage-grouse and each marker type

Population	# Samples		2004 Pop. Est.
	MSAT	SNP	
Cimarron	4	4	74
Crawford	21	12	157
Dove Creek	43	12	98
Gunnison Basin	116	12	3978
Piñon Mesa	19	10	182
Poncha Pass	0	0	10
San Miguel	51	10	206

*MSAT* microsatellites, *SNP* single nucleotide polymorphisms. Population estimates of the 2004 population size = 2004 Pop. Est. [70]

The double digestion RAD-Seq approach to reduced representation sequencing is one of the most commonly used genomic library preparations to generate SNP genotypes. With the increasing use of RAD-Seq generated SNPs instead of microsatellite data for conservation questions and monitoring, here we aim to compare population genetic parameters specifically from RAD-Seq generated SNPs and microsatellites. To date, few studies have compared the consequences of marker types in conservation objectives when a typical RAD-Seq protocol is used (though see [34, 45, 50, 51, 64]). Given the impact of decisions made during RAD-Seq protocols on downstream analysis [72], the prevalence of RAD-Seq generated SNP datasets, and the limited empirical examples of comparisons to more traditional microsatellite analyses, more comparisons can provide insight into the limitations or benefits of RAD-Seq generated SNP data and the future utility of microsatellite loci. Through previous studies on this species of conservation concern, we had access to two range-wide Gunnison sage-grouse genetic datasets, one of microsatellite [73] and one of SNP loci [65]. We had three specific objectives in this study: (1) compare genetic diversity metrics across datasets, (2) compare genetic differentiation metrics across datasets, and (3) compare clustering methods across datasets and investigate evidence of evolutionary independence among populations.

## Results

### Genetic diversity

**Fig. 2:**
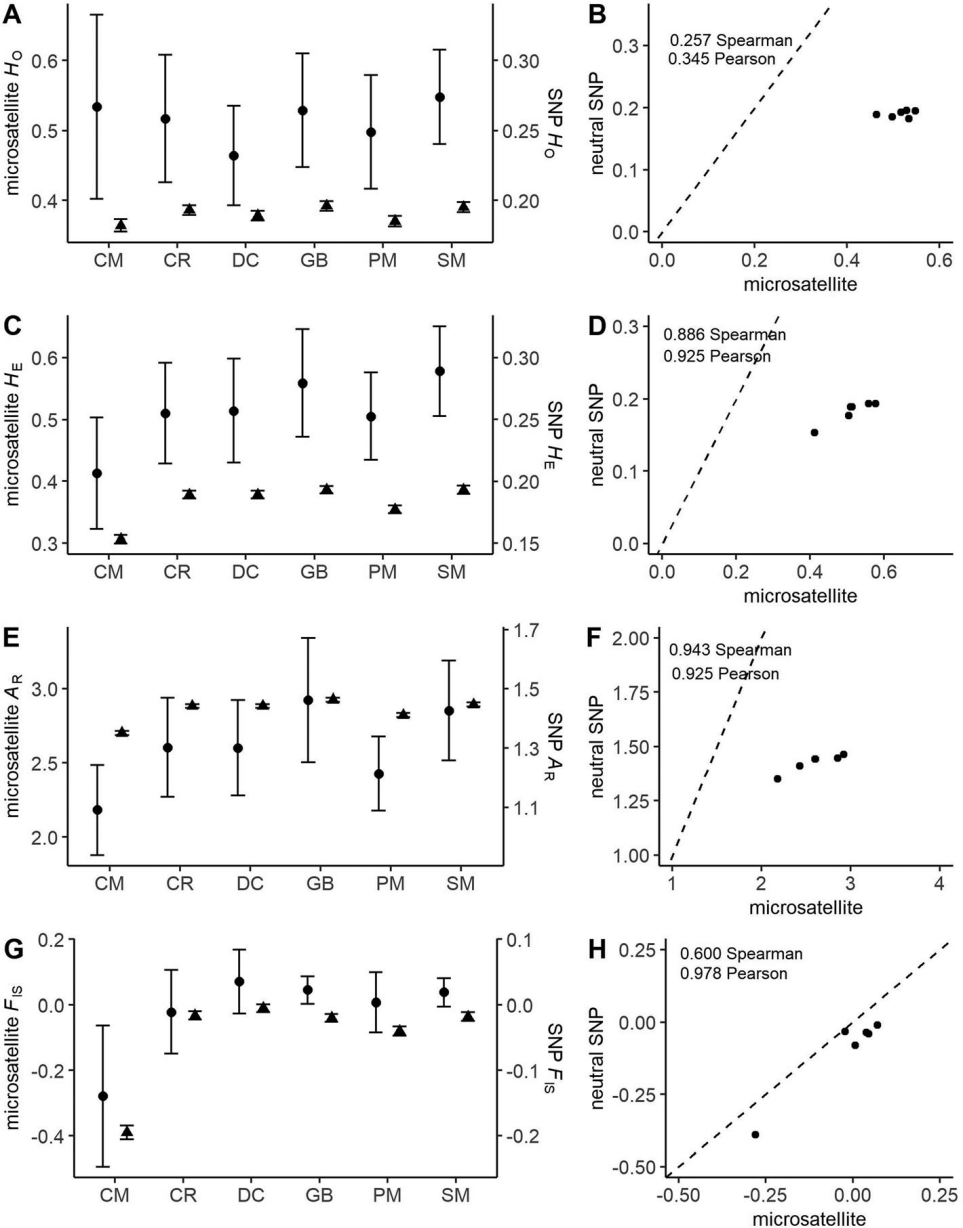
Comparison of genetic diversity values for Gunnison sage-grouse populations. Confidence intervals (95%) around mean values for microsatellite (●) and putatively neutral SNP (▲) loci were constructed. Estimates for observed heterozygosity (*H*_O_; **a**), expected heterozygosity (*H*_E_; **c**), allelic richness (*A*_R_; **e**), and inbreeding coefficient (*F*_IS_; **g**) are shown in the left-hand column. Populations are abbreviated along the x-axis: CM = Cimarron, CR = Crawford, DC = Dove Creek, GB = Gunnison Basin, PM = Piñon Mesa, SM = San Miguel. Relationships between estimates from microsatellites and SNPs for *H*_O_ (**b**), *H*_E_ (**d**), *A*_R_ (**f**) and *F*_IS_ (**h**) are shown in the right-hand column. Spearman rank and Pearson’s correlation coefficient are also included in the plots in the right-hand column. Dashed line corresponds to a 1:1 relationship

For all diversity metrics, 95% confidence intervals calculated from SNPs were narrower than confidence intervals from microsatellites (Fig. 2; Additional file 1: Table S1). Microsatellite estimates had large confidence intervals in all cases, which resulted in no significant differences among population estimates. In contrast, the narrower confidence intervals with SNPs resulted in significant differences between populations. Of the four metrics, *H*_O_ had the lowest correlation across marker type (Spearman *ρ* = 0.257, Pearson *r* = 0.345) (Fig. 2b). As theoretically expected, values of *H*_O_ from microsatellites in all populations were ~ 0.500 (range: 0.464–0.548) while values from SNPs were lower, ~ 0.200 (range: 0.183–0.197; Fig. 2a). However, both marker types resulted in relatively consistent population ranks based on mean *H*_O_ (*p* = 0.031, Wilcoxon paired signed-rank). Values of *H*_E_ showed high correlation (Spearman *ρ* = 0.886, Pearson *r* = 0.925), and relative consistency in ranking populations across marker types (*p* = 0.031, Wilcoxon paired signed-rank). The values for *H*_E_ were within similar ranges as *H*_O_, microsatellite estimates at ~ 0.500 (range: 0.413–0.578) and SNP estimates at ~ 0.200 (range: 0.154–0.194; Fig. 2c and d). Similarly, allelic richness showed high levels of correlation (Spearman *ρ* = 0.943, Pearson *r* = 0.925), and consistent ranking of populations by levels of genetic diversity (*p* = 0.031, Wilcoxon paired signed-rank) across marker type (Fig. 2e and f). Estimates of *F*_IS_ also showed relatively high correlation (Spearman *ρ* = 0.600, Pearson *r* = 0.978), however, ranking of populations was not as consistent (*p* = 0.563, Wilcoxon paired signed-rank) across marker types and the magnitude of the values for each marker type resulted in different inferences in some cases (Fig. 2g and h); microsatellites indicated outbred (minimum value: − 0.279) to slightly inbred (maximum value 0.071) populations while SNPs indicated slightly to moderately outbred populations (− 0.194 – − 0.004).

### Genetic differentiation

**Fig. 3:**
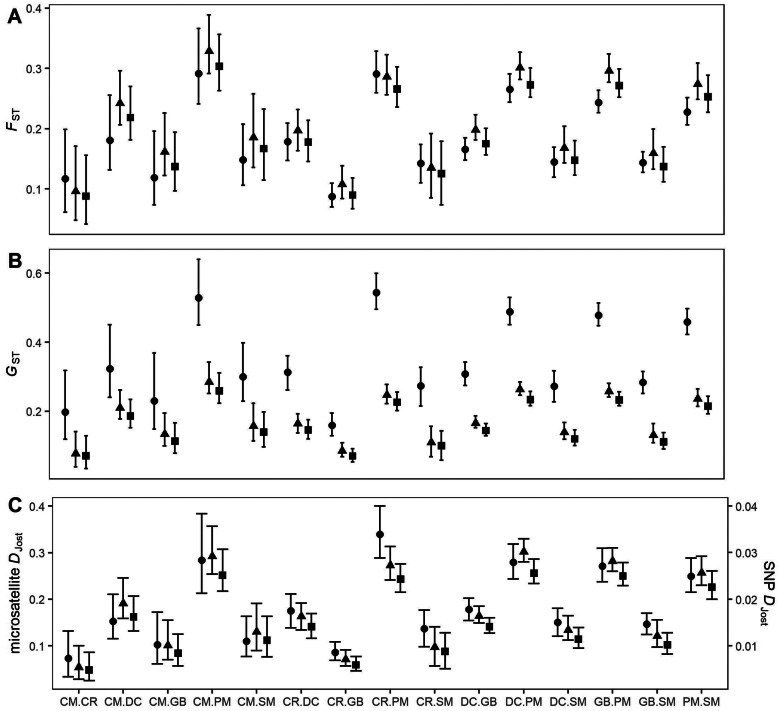
Comparison of genetic differentiation values for pair-wise comparisons of Gunnison sage-grouse populations. Confidence intervals (95%) around mean values for microsatellite (●), putatively neutral SNP (▲), and all SNP (■) loci. Pair-wise estimates are for *F*_ST_ (**a**), *G*_ST_ (**b**), and *D*_Jost_ (**c**). Populations in pair-wise comparisons are abbreviated along the x-axis: CM = Cimarron, CR = Crawford, DC = Dove Creek, GB = Gunnison Basin, PM = Piñon Mesa, SM = San Miguel; CM.CR = F_ST_ between Cimarron and Crawford

**Fig. 4:**
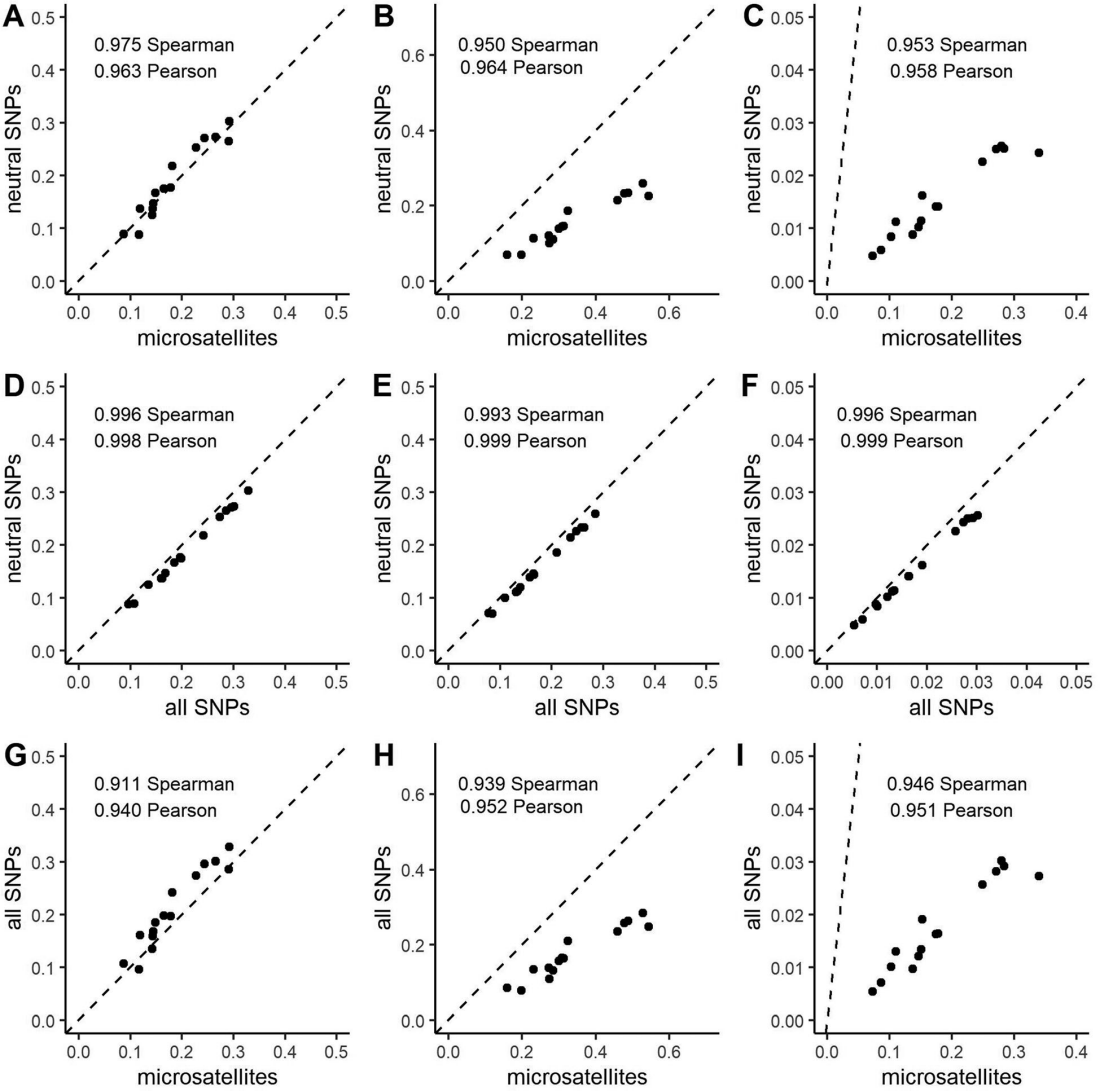
Correlation of differentiation metrics for Gunnison sage-grouse populations. Relationships between estimates from different datasets: microsatellites, putatively neutral SNPs, and all SNPs for *F*_ST_ (**a**,**d**,**g**), *G*_ST_ (**b**,**e**,**h**), and *D*_Jost_ (**c**,**f**,**i**) are shown in respective panels. Axes are labeled by dataset. Spearman rank and Pearson’s correlation coefficient are included in the upper left-hand corner of each panel. Dashed line corresponds to a 1:1 relationship

Generally, genetic differentiation estimates from SNP datasets had narrower confidence intervals in comparison to estimates from microsatellites (Fig. 3; Additional file 1: Table S2) which were significantly correlated in all pair-wise comparisons (Mantel *r* > 0.9, *p* < 0.001). All differentiation metrics had a high correlation across marker types and datasets (Fig. 4). For *F*_ST_ and *G*_ST_, confidence intervals for population estimates from microsatellites and both SNP datasets typically overlapped (Fig. 3a and b). Estimates of D_Jost_ from microsatellites and both SNP datasets did not overlap and the magnitude of microsatellite estimates were consistently much higher in comparison to SNP estimates (Fig. 3c), though the same general pattern remained (Fig. 4c and i). Similarly, values of *G*_ST_ estimated with microsatellites were also larger in magnitude than with SNPs, though to a lesser degree than observed with *D*_Jost_ (Fig. 3b).

### Clustering

**Fig. 5:**
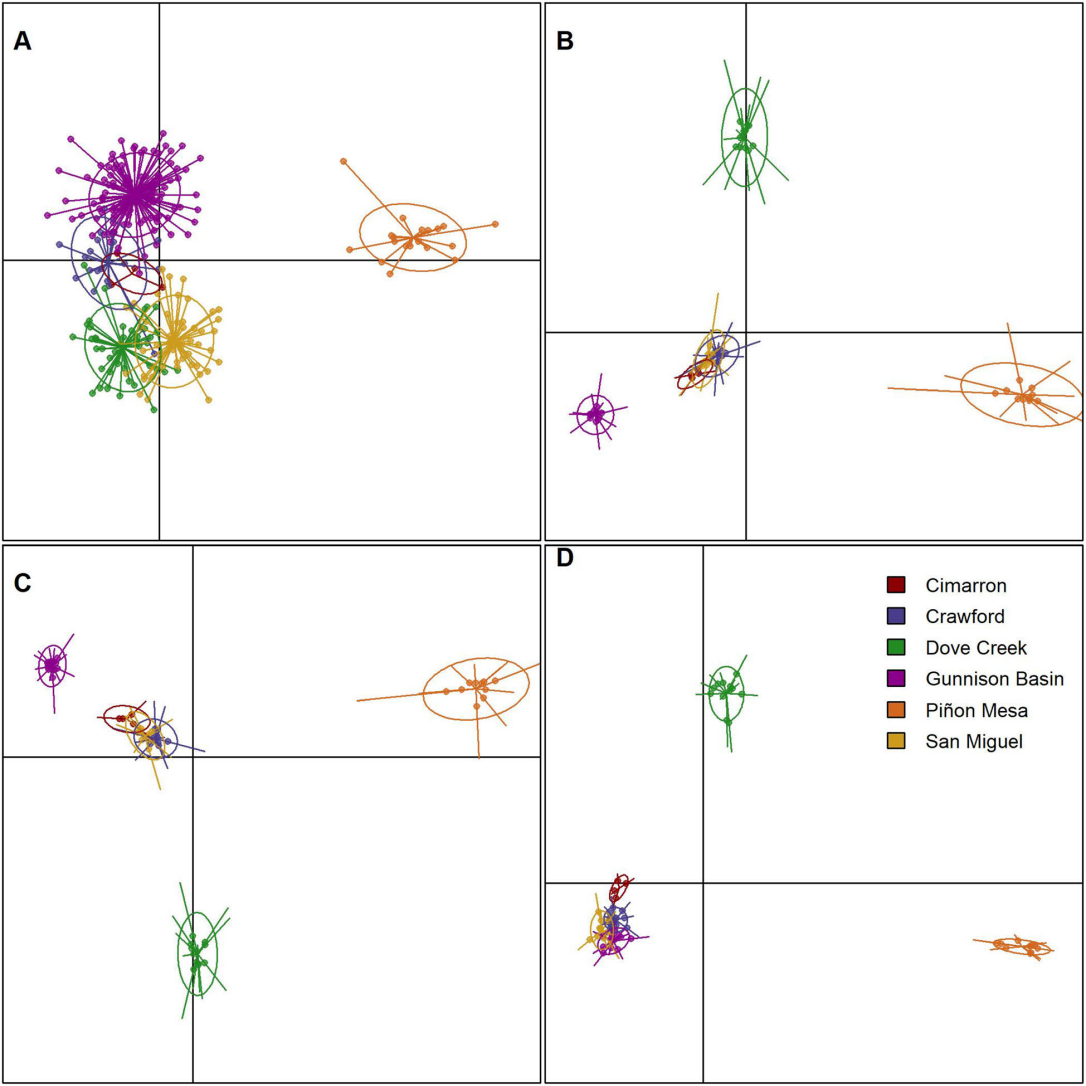
Star-plots of DF1 (x-axis) and DF2 (y-axis) from discriminant analysis of principle components (DAPC) for Gunnison sage-grouse. Panels correspond to different datasets: **a** microsatellite, **b** all SNPs, **c** putatively neutral SNPs, **d** and candidate adaptive SNPs. Each point represents an individual color coded by sampling origin

The lowest BIC for hypothetical genetic clusters in DAPC corresponded to 6 groups with microsatellites (BIC = 484.603), 5 groups with all SNPs (BIC = 454.768), and putatively adaptive SNPs (BIC = 298.376), and 4 with putatively neutral SNPs (BIC = 449.803). The optimal number of PCs to include in the DAPC analysis as determined by the a-score method was 22 for microsatellites, 6 for all SNPs, 5 for putatively neutral SNPs, and 6 for putatively adaptive SNPs. Clustering of individuals in DAPC with microsatellites identified Piñon Mesa as the only population that clearly separates from the other populations along discriminant function 1 (Fig. 5a), while discriminant function 2 pulls populations into identifiable groups though still with overlap (Fig. 5a). With all and putatively neutral SNPs, discriminant function 1 separates Gunnison Basin and Piñon Mesa from the other populations (Fig. 5b and c), and discriminant function 2 separates Dove Creek (Fig. 5b and c). The candidate adaptive loci dataset shows Piñon Mesa and Dove Creek clearly separated along discriminant function 1, while San Miguel, Cimarron, Crawford, and Gunnison Basin cluster together (Fig. 5d).

**Fig. 6:**
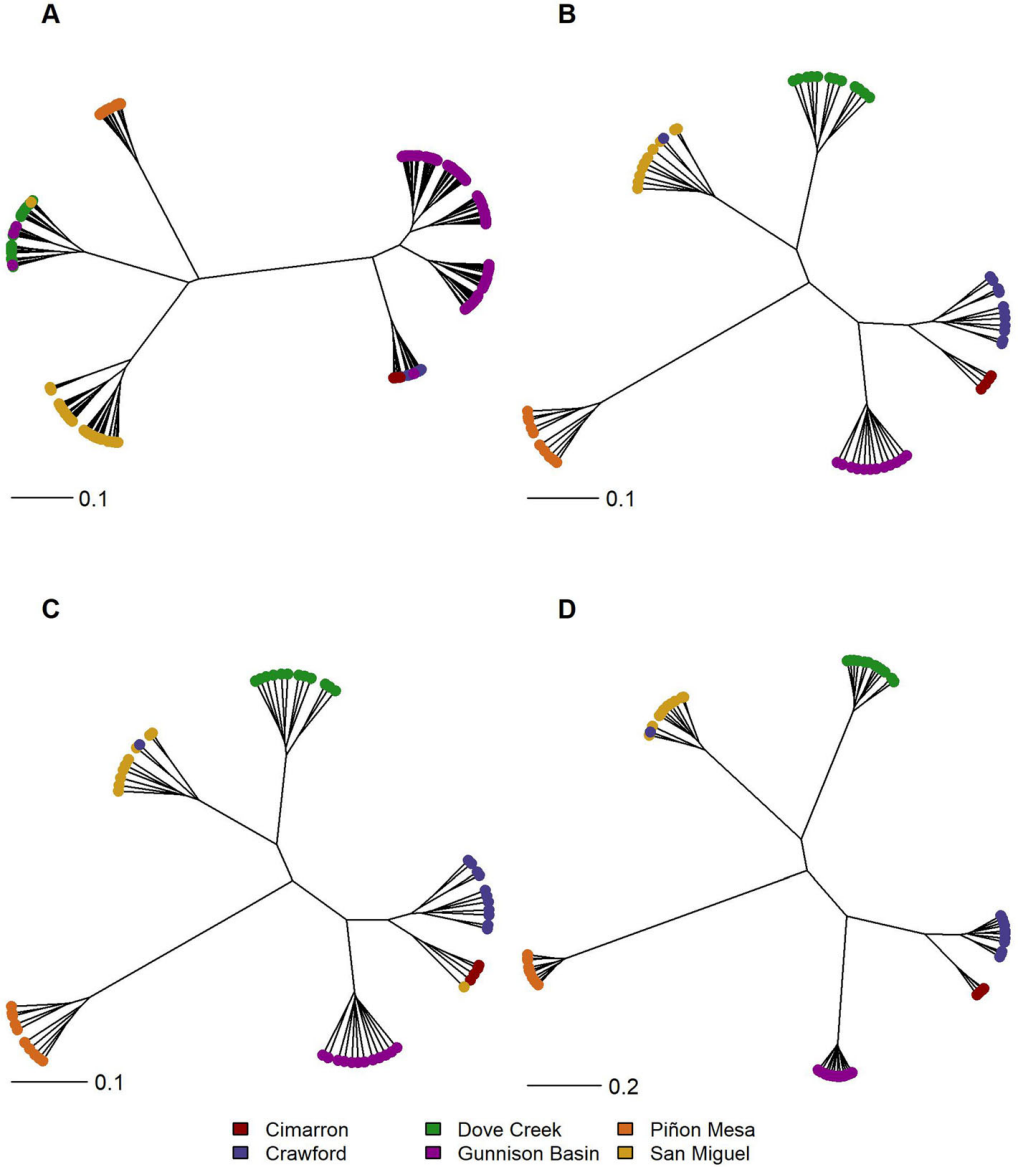
Comparison of dendrograms of individual Gunnison sage-grouse using the hierarchical clustering method “ward. D2”. Panels correspond to different datasets: microsatellites (**a**), all SNPs (**b**), putatively neutral SNPs (**c**), and candidate adaptive loci (**d**). Colors indicate sampling origin

The dendrogram created from microsatellite data generally grouped individuals into known populations where Cimarron, Crawford, and Gunnison Basin grouped closest together with Piñon Mesa, Dove Creek, and San Miguel grouping closer together but away from the Cimarron, Crawford, Gunnison Basin individuals (Fig. 6a). Cimarron and Crawford individuals were grouped together on a single branch, along with two Gunnison Basin individuals. Additionally, two individuals from Gunnison Basin and an individual from San Miguel cluster with the Dove Creek individuals. Similar to the clustering pattern observed in DAPC, all SNPs and putatively neutral SNPs resulted in nearly indistinguishable grouping patterns where all populations are identifiable on individual branches (Fig. 6b and c, respectively). With both the all SNP and putatively neutral SNP datasets Cimarron, Crawford, and Gunnison Basin group most closely, Piñon Mesa is the most distant from the center, and a single individual sampled in Crawford grouped with the San Miguel individuals. With neutral SNPs a single San Miguel individual grouped with Cimarron (Fig. 6c). Though similar to the other SNP dendrograms in that samples clustered into distinct populations, branch lengths appear somewhat longer in the candidate adaptive loci dataset (Fig. 6d). When considering hierarchical clustering using methods in addition to “ward. D2”, the patterns are generally similar though some differences are notable, particularly when comparing the results of microsatellites to any of the SNP datasets. The “single” method, which bases branch length between groups on the closest individual in each group, does not result in distinct populations using microsatellite data (Additional file 1: Fig. S1A), but results in the same clustering pattern as the “ward. D2” method for all SNPs (Additional file 1: Fig. S1B), putatively neutral SNPs (Additional file 1: Fig; S1C), and candidate loci (Additional file 1: Fig. S1D). The “complete” method, which bases branch length between groups on the most distant individuals, shows Cimarron, Crawford, and San Miguel individuals nested between groups of Gunnison Basin individuals while Dove Creek and Piñon Mesa are distinct when using microsatellites (Additional file 1: Fig. S2A), but results in nearly the same clustering pattern as with “ward. D2” when using all SNPs (Additional file 1: Fig. S2B), putatively neutral SNPs (Additional file 1: Fig. S2C) and candidate adaptive loci (Additional file 1: Fig. S2D), though a single San Miguel individual clusters with Cimarron using all SNPs and putatively neutral SNPs (Additional file 1: Fig. S2B and S2C).

## Discussion

In general, we found that measures of diversity and differentiation generated from microsatellite and SNP data were typically in agreement in ranking of population estimates, although magnitudes of estimates were quite different. Metrics of differentiation, however, had consistently higher correlation than most metrics of diversity. Our results also confirmed that increased numbers of SNP loci can dramatically reduce the confidence intervals for mean estimates, increasing precision, although this was not true for all differentiation measures. We also demonstrated that clustering of individuals for the purpose of identifying evolutionarily or demographically distinct units can be variable depending on clustering method used and marker type.

### Genetic diversity

Of the four diversity metrics evaluated here, *H*_E,_*F*_IS,_ and *A*_R_ were the metrics with the highest correlation between microsatellites and SNPs (see Fig. 2). *H*_O_, however, showed relatively low correlation across marker types. Several previous studies with variable numbers of markers, sample sizes, and SNP discovery approaches generally agree with our correlation of diversity metrics across marker types, though positive relationships were sometimes moderate [43, 45, 51, 58, 59, 64]. None of these studies report *H*_O_, and so there was no comparison for our relatively low correlation across marker types for this metric. Still, some argue higher correlation may be influenced by the number of SNPs [43], or whether loci represent a high proportion of the genome-wide polymorphism [74, 75]; two aspects of SNP datasets that will vary by study and may play a role in our observed correlations. Importantly, similar to findings by Fischer et al. [59], the high variance in microsatellite data for all diversity metrics resulted in almost no significant difference between populations; differences that were detected with SNPs. Though we, like others, show high correlation among marker types, the increased precision in estimates allow distinction of populations when using SNP data. For general monitoring of changes in diversity for conservation or management of a species, either marker type would prove useful. This was not necessarily true for *F*_IS_, where generally high correlation was observed, though ranking was not as consistent, and SNPs failed to detect the indication of inbreeding that was apparent with microsatellites (i.e., *F*_IS_ > 0; Fig. 1 & Table S1). It is worth restating that samples were originally selected for the SNP data based on relatedness values estimated with the microsatellite data. Logically, selecting minimally related individuals could result in the SNP data producing F_IS_ estimates consistent with more outbreeding than the original full microsatellite dataset. However, when we compared diversity metrics from a reduced microsatellite dataset including only the individuals used in the SNP dataset, we find no significant differences for any diversity metric according to the confidence intervals (Table S1). However, the mean F_IS_ estimates for the reduced microsatellite dataset would suggest a change in the sign of the estimate (i.e., either from inbreeding to outbreeding or outbreeding to inbreeding) for three populations. SNPs, however, would have an obvious advantage if conservation actions required an understanding of the relative levels or ranking of most measures of genetic diversity.

### Genetic differentiation

In general agreement with multiple studies [21, 45, 58–60, 64] all metrics of differentiation showed high correlation between microsatellites and SNP datasets, with correlation coefficients greater than 0.90 in all tests (Fig. 4) and significant Mantel correlations (Mantel *r* > 0.9, *p* ≤ 0.05 for all comparisons). Some argue reliance on a single measure of differentiation for conservation purposes risks inaccurate characterization of populations [76–78]. Our findings, however, echo other empirical examples where different metrics result in the same inference [11, 60], but only when ranking populations (Wilcoxon paired signed-rank test *p* ≤ 0.05 in all comparisons). Different metrics of population differentiation showed a consistent pattern of which populations were most similar, though the magnitude of a metric was sometimes very different. If the magnitude of the differentiation metric is of conservation relevance, then the marker types are not equivalent.

The appropriateness of a differentiation metric in conservation can be further impacted by additional differences in marker types. The different metrics measure different things. *D*_Jost_ is considered a relative degree of allelic differentiation, while *F*_ST_ and *G*_ST_ are fixation indices [78]. Similar to some previous studies, we found *D*_Jost_ tended to produce values higher in magnitude with microsatellites than with SNPs [64]. Each microsatellite locus will always have higher per locus allelic diversity than biallelic SNP loci, and therefore result in higher magnitude estimates of *D*_Jost_ [78, 79]. We also found higher *G*_ST_ estimates with microsatellites (Fig. 3b & Table S2), although the magnitude of difference between the values calculated from different marker types was not as dramatic as that with *D*_Jost_ (Fig. 3c & Table S2). *G*_ST_ depends on heterozygosity, so the higher theoretical maximum heterozygosity for microsatellite loci (*H*_E_ = 1), versus the theoretical maximum heterozygosity for SNP loci (*H*_E_ = 0.5), will also result in higher magnitudes of *G*_ST_ when calculated from microsatellite data. Importantly, *F*_ST_ has proven to be more robust to per locus allelic diversity and heterozygosity (though see [59, 60]). From a conservation perspective, *F*_ST_ may prove most useful in the transition from microsatellite loci to SNPs for genetic monitoring because of the observed consistency across marker types. However, *D*_Jost_ may actually be the more relevant conservation metric if comparing relative degrees of differentiation or identifying isolated groups because the level of differentiation takes into account allelic identity and not just population level fixation [78]. Fixation indices will indicate two populations fixed for the same allele are distinct because both populations lack diversity at a locus while allelic differentiation metrics will not because the identity of the fixed allele is the same and therefore populations are not different at that locus.

The three differentiation metrics evaluated here also have different sensitivities to the underlying mutation rate generating each type of marker with *F*_ST_ proving more robust [78, 80]. In addition to impacts from mutation rate on different metrics, migration rate and population size are also important to consider. Whitlock [80] demonstrated that for low mutation rates, approximately that of SNPs (10E-9), and low migration rates among a small number of populations, measures of differentiation as measured by *D*_Jost_ will be much smaller in magnitude, and to a lesser degree, so will *G*_ST_. Relatively low levels of mutation, migration, and small populations typically correspond to lower allelic diversity. Gunnison sage-grouse is composed of seven isolated populations, with very low migration rates among populations, though not as low as SNP mutation rates. Our comparison of *F*_ST_ values across marker types demonstrates relatively consistent agreement between both magnitude and ranking of pair-wise comparisons (Fig. 3a). As predicted, *D*_Jost_ and *G*_ST_ consistently rank comparisons across marker type, though the magnitude of metrics were lower for SNPs than microsatellites; much lower in the case of *D*_Jost_ (Fig. 3b and c). Overall, our results empirically demonstrate the predicted impact population configurations can have on measures of differentiation.

Both marker types suffer from additional characteristics that can influence estimates of differentiation. In addition to the influence of heterozygosity and allelic diversity on measures of differentiation, there is a trade-off between the number of loci and the per locus information content. More SNP loci will be required to obtain the same resolution in differentiation metrics from fewer microsatellite loci, because the number of alleles per locus can impact the ability to detect reproductively isolated groups. If a locus only has two alleles, as is typical with SNP loci, the chances of populations differing in allele frequencies at high enough levels to detect isolation is lower. Conversely, if a locus has multiple alleles shared among populations, the differences in allele frequencies are more likely to be detected, therefore showing the level of reproductive isolation. Many studies have provided suggestions on the number of SNPs required to obtain resolution in differentiation comparable to that obtained with microsatellites, ranging from two to 11 times more SNP loci [25, 57, 81]. However, more recent work has indicated fewer SNPs than previously suggested can be sufficient [47, 58, 60, 82]. We did not explicitly evaluate the number of SNP loci required to obtain estimates with the precision of microsatellites, though we do demonstrate that 14,091 biallelic putatively neutral SNPs results in comparable estimates to 22 microsatellites with three to 18 alleles per locus. Our study likely reflects a typical number of SNPs which would be obtained with a RAD-Seq protocol, the most commonly used approach for wildlife species. We therefore, demonstrate RAD-Seq generated SNP genotypes can produce comparable differentiation estimates to those obtained with microsatellites.

We do not, however, demonstrate a dramatic reduction in confidence intervals around those measures of differentiation (Fig. 3), as has been predicted. However, in pair-wise comparisons of differentiation, the small sample size of one of the populations is known to impact the confidence intervals [83]. In our data, we see this trend particularly for *F*_ST_ and with comparisons involving our smallest population represented by the fewest samples, Cimarron (Fig. 2). In species of conservation concern variable population sizes are often unavoidable, and by increasing the number of loci sampled (> 1000 SNPs) robust estimates of differentiation can still be obtained [83].

### Clustering

Contrary to our findings for differentiation, the clustering analyses showed an increase in precision with SNP data that is consistent with previous studies [45, 61, 64]. We used multiple methods to cluster individuals (dendrograms and DAPC) all of which showed general agreement of clustering by population of origin (Figs. 5b, c, and d, 6b, and c). The SNP data, however, resulted in tighter groups of individuals (Figs. 5c and 6c) relative to the somewhat loose clusters of individuals with microsatellite data (Figs. 5a and 6a). The number of individuals sampled varied by marker type in our study (256 in the microsatellite dataset versus 60 in the SNP datasets), which could potentially contribute to the lower resolution in clustering analyses when compared to the SNP data. However, when we looked at clustering of microsatellite data using only the 60 individuals included in the SNP data, the patterns of clustering remain the same (Additional file 1: Fig. S3 and S4), similar to what Lemopoulos et al. [45] found.

The potential impact of conservation actions on a species local fitness and how that relates to adaptive divergence is important to consider [84], especially for a species with geographically distinct and declining populations. Identifying candidate adaptive loci can provide insight into the potential adaptive divergence among populations and the potential for local adaption. We also compared clustering of individuals by previously identified candidate adaptive loci [65], an objective that cannot be accomplished with microsatellite loci (i.e., neutral loci) alone. We found evidence of adaptive divergence in two or three populations (Figs. 5d, 6d, S1D, and S2D), depending on the method used for clustering. Though the small populations and small sample sizes could be causing fixation of alleles due to strong drift, the approaches used to identify candidate adaptive loci generally control for demography (e.g., BayPass and partial RDA; see Zimmerman et al. [65]).

Comparing the clustering of individuals with all SNP loci (Figs. 5b and 6b) to clustering including only putatively neutral (Figs. 5c and 6c) or candidate adaptive (Figs. 5d and 6d) loci, we see that neutral genetic processes in Gunnison sage-grouse may be stronger than adaptive divergence. Evidence of adaptive divergence corresponded to approximately 6% (942 SNPs) of the sampled genome. Neutral and adaptive variation are both important to consider for designation as an ESU or conservation unit [37, 40]. However, the ratio of neutral versus adaptive loci undoubtedly influences identification of distinct units. In addition to considering the marker type, it could be important to identify what proportion of the genome must hold the signal for adaptive divergence for formal designation. The term functionally significant unit (FSU) was recently suggested to describe conservation units based on ecologically important genes [85]. More recently, a single ecologically important gene was used to propose conservation units for salmon [12]. Most genes underlying phenotypes are quantitative in nature with only preliminary ecological links, and so single gene definitions of conservation units will be rare at best [86]. Further, the focus on identifying conservation units based on potential adaptive divergence may result in unintended consequences such as reduced effort to conserve or restore habitat [86], overlooking the role vicariance events may play in adaptation [87], or a failure to acknowledge traits that are adaptive in a given environment presently may not be locally adapted in future environments. Importantly, questions of local adaptation and evolutionary independence cannot be considered with microsatellite loci, or any neutral loci alone. Attempts to identify distinct units with genetic data should focus on using SNP data, or a combination of neutral (microsatellite or SNP) in combination with known ecologically important functional regions.

## Conclusions

We demonstrated that RAD-Seq generated SNPs from a non-model organism are generally comparable to microsatellites for measuring population genetic parameters, in agreement with some previous studies [45, 51, 64]. The rapid progression away from use of microsatellites and toward use of SNP data in conservation and management applications highlights the importance of these types of comparisons and calls into question the future usefulness of microsatellite data. As we, and others, have shown, the same general inference can typically be drawn about population-level genetic differentiation and diversity, irrespective of marker type. However, we showed that SNPs had three main advantages over microsatellites. First, the much smaller confidence intervals around diversity measures allowed distinctions between populations to be made with SNP data. From a conservation perspective, all populations of Gunnison sage-grouse would have been considered equally diverse using microsatellite loci, while there were clear differences in relative diversity with SNP data. Second, clustering methods showed a dramatic increase in the power to separate individuals into distinct groups. Microsatellite data failed to clearly separate individuals into populations in nearly all instances; populations that were clearly differentiated with SNP data. Third, SNP data allows consideration of local adaptation.

We also further demonstrated the impact of marker choice on differentiation metrics—different marker types resulted in very different magnitudes. This finding exemplifies the dangers of using thresholds for differentiation and diversity metrics for conservation objectives. If the magnitude of the value is not of importance, all metrics except *H*_O_ and *F*_IS_ were able to consistently rank populations or population pairs across marker types in our study. While we found clear advantages for use of SNPs in population genetics, there remain some limitations at present. Primarily, generation of SNP datasets requires relatively large quantities of high quality DNA, which is often difficult to obtain from species of conservation concern. However, investing in the development of a SNP panel or using a target capture approach can facilitate use of low quality samples [88]. On the other hand, microsatellites are extremely useful with low quality samples, are becoming less costly and time consuming to develop (e.g., Castoe et al. [89]), and have already been widely used in conservation and management programs for long-term monitoring of many species. Although general usefulness of microsatellites in the future is uncertain, microsatellite loci will likely remain useful for relatedness, parentage analysis, and genetic mark-recapture due to their highly polymorphic nature and mixed performance with SNP data [46, 47, 49, 53, 54, 88].

## Methods

### Data

#### Microsatellite genotypes

Blood samples were collected near breeding grounds within six of the populations as part of a 2005 study [69]. The dataset we use here is composed of 254 individuals from these previously collected samples that were genotyped at a larger set of microsatellite loci for a 2019 study [73]. Sample size varied by population: Cimarron = 4, Crawford = 21, Dove Creek = 43, Gunnison Basin = 116, Piñon Mesa = 19, San Miguel = 51. Populations are named after nearby Colorado towns with 2 exceptions, Piñon Mesa is located west of Grand Junction and San Miguel is south of Norwood. We amplified 22 grouse-specific microsatellite loci using the Polymerase Chain Reaction (PCR) and with the components and concentrations described in Oyler-McCance and Fike [90] with thermal profiles and annealing temperatures as originally published. The microsatellite primers used included: MSP11, MSP18, reSGCA5, reSGCA11, SG21, SG23, SG24, SG28, SG29, SG30, SG31, SG33, SG36, SG38, SG39, SGCTAT1, SGMS06.4, SGMS06.8, TTT3, TUT3, TUT4, and WYBG6 [91–96]. See Zimmerman et al. [73] for details on DNA extraction and genotyping. The final microsatellite dataset was composed of 22 relatively polymorphic sampled loci, for a total of 254 individuals, with variable representation by geographic population.

#### Single nucleotide polymorphism (SNP) genotypes

From the same 254 previously collected blood samples that were genotyped at microsatellite loci, a subset were previously chosen for RAD-Seq [65] based on two criteria: population of origin and relatedness. The goal was to obtain an equal number of minimally related individuals from each population. The exception to these requirements was the Cimarron population, which only had four samples; consequently all Cimarron samples were included. These criteria for sample selection were necessary because of limited available funding and high enough quality samples. See Zimmerman et al. [65] for details on RAD-Seq library preparation and bioinformatics. The complete SNP dataset was composed of 15,033 loci across 35 “pseudo-chromosomes” (chromosome scaffolds inferred from synteny with chicken) for 60 individuals (Cimarron = 4, Crawford = 12, Dove Creek = 12, Gunnison Basin = 12, Pinon Mesa = 10, San Miguel = 10). A putatively adaptive SNP dataset composed of all 942 loci that were previously identified as potentially under selection in outlier locus analyses and genotype-environment association analyses was also created. Methods used to identify putatively adaptive loci included BayPass [97], pcadapt [98], and a redundancy analysis as described in [99]. Environmental covariates used in the genotype-environment association included average spring precipitation, average fall precipitation, spring maximum temperature, winter maximum vapor pressure deficit, compound topographic index (a proxy for soil moisture), green-up rate (a measure of the progression of the growing season), big sagebrush cover, and a dryness index (see Zimmerman et al. [65] for details on loci under selection). A putatively neutral SNP dataset was created by excluding all putatively adaptive loci. The final putatively neutral SNP dataset included 14,091 biallelic loci across 34 pseudo-chromosomes, for 60 individuals with relatively equal representation from each geographic population.

### Analysis of genetic diversity

For each putatively neutral dataset, we estimated observed heterozygosity (*H*_O_), expected heterozygosity (*H*_E_), allelic richness per locus (*A*_R_), and inbreeding coefficient (*F*_IS_) using the ‘diveRsity’ [100] package in R [101]. Diversity metrics were estimated for each locus based on 1000 bootstraps and reported as a mean and 95% confidence intervals constructed from the standard deviation across all loci. Mean allelic richness per locus was also estimated with rarefaction for comparison (results included in Additional file 1: Table S1). Diversity metrics were calculated for both datasets and used to compare estimates from microsatellite and putatively neutral SNPs. Pearson and Spearman rank correlation coefficients were estimated to evaluate congruence for all paired metrics. Wilcoxon paired signed-rank test in the R package ‘MASS’ [102] was used to evaluate the consistency of ranked values among datasets.

### Analysis of genetic differentiation

For genetic differentiation we compared analysis results from microsatellites, all SNPs, and putatively neutral SNPs. We used the ‘diveRsity’ package in R to calculate *F*_ST_ [103] with confidence intervals based on 1000 bootstraps. Because there is concern about comparing pair-wise *F*_ST_ values when using loci with variable levels of heterozygosity, we also calculated pair-wise *G*_ST_ [104] and *D*_Jost_ [79] with confidence intervals based on 1000 bootstraps. *D*_Jost_ differs from both *F*_ST_ and *G*_ST_ in that it is a measure of the fraction of allelic variation among populations and is not constrained by the expected level of heterozygosity within the subpopulation [79]. Significance of correlation between pair-wise differentiation measures for each dataset was evaluated with the Mantel *p*-value as calculated with the ‘vegan’ R package [105].

### Analysis of clustering

We compared the identification of distinct units using microsatellites, all SNPs, putatively neutral SNPs, and putatively adaptive SNPs. First, we performed discriminnant analysis of principal components (DAPC) with microsatellites, putatively neutral SNPs, all SNPs, and candidate adaptive loci with the ‘adegenet’ package in R [106]. DAPC summarizes genotypes in principal components (PC) that are then used to construct linear functions that simultaneously maximize among-cluster variation and minimize within cluster variation. We used the *K*-means clustering algorithm and identified the number of genetic clusters based on the Bayesian Information Criterion (BIC). We retained all of the PCs, ran the algorithm for 100,000 iterations, and used 10 starting centroids per run. The number of genetic clusters (*K*) with the lowest BIC was selected, as recommended by Jombart et al. [107]. After we identified optimal *K* for each dataset, we used the a-score method to identify the optimal number of PCs to retain in DAPC while constructing linear functions to describe genetic differentiation among *K* groups. Second, we created dendrograms from an individual-based genetic distance matrix calculated as the proportion of differing nucleotide sites [108], excluding missing data in pair-wise estimations, with 1000 bootstraps for each dataset. We used the hierarchical clustering algorithm *hclust* in R and the “ward. D2” method [109]. The “ward. D2” method minimizes the total within cluster variance and minimizes information loss associated with each cluster. For comparison of hierarchical clustering methods we also included dendrograms created with a more conservative method tending to form loose groups, sometimes prematurely (“single” method; Additional file 1: Fig. S1) and a more relaxed method tending to form tighter and smaller groups (“complete” method; Additional file 1: Fig. S2). For comparison, results for clustering analyses with a reduced microsatellite dataset using only individuals in the SNP dataset are included in the supplemental materials (Additional file 1: Fig. S3 and Fig. S4).

## Supplementary information

**Additional file 1: Table S1.** Diversity statistics. **Table S2.** Differentiation statistics. **Fig. S1.** Dendrograms created using the “single” (based on closest pair) method. **Fig. S2.** Dendrograms created using the “complete” (based on furthest pair) method. **Fig. S3.** Dendrograms created using microsatellite loci from the 60 individuals included in the SNP dataset. **Fig. S4.** Discriminant analysis of principle components (DAPC) for microsatellite from the 60 individuals sampled for SNPs.

## Data Availability

The microsatellite dataset is available in the U.S. Geological Survey Science Base repository, 10.5066/P920WO0Q. Genomic sequencing data for this study were deposited in GenBank (biosample accession numbers: SAMN10844489-SAMN10844548) and SNP genotypes for this study were deposited in the U.S. Geological Survey ScienceBase, 10.5066/P94ET592.

## Abbreviations

BIC: Bayesian information criterion
CM: Cimarron
CR: Crawford
DAPC: Discriminant analysis of principal components
DC: Dove Creek
DNA: Deoxyribonucleic acid
ESA: Endangered species act
ESU: Evolutionarily significant unit
FSU: Functionally significant unit
GB: Gunnison Basin
MSAT: Microsatellite
PC: Principal component
PCR: Polymerase chain reaction
PM: Piñon Mesa
SM: San Miguel Basin
SNP: Single nucleotide polymorphism
