# Supplementary material for: An empirical comparison of population genetic analyses using microsatellite and SNP data for a species of conservation concern
Source: BMC Genomics. 2020 Jun 1;21:382. doi: 10.1186/s12864-020-06783-9 (PMC7268520; doi:10.1186/s12864-020-06783-9)
Supplement: Supplementary file 1 — Additional file 1: Table S1. Diversity statistics. Table S2. Differentiation statistics. Fig. S1. Dendrograms created using the “single” (based on closest pair) method. Fig. S2. Dendrograms created using the “complete” (based on furthest pair) method. Fig. S3. Dendrograms created using microsatellite loci from the 60 individuals included in the SNP dataset. Fig. S4. Discriminant analysis of principle components (DAPC) for microsatellite from the 60 individuals sampled for SNPs. [file 12864_2020_6783_MOESM1_ESM.docx]

Table S1. Diversity statistics for Gunnison sage-grouse populations using different genetic marker types. Means and SEs for all diversity metrics (H_O_ = observed heterozygosity, H_E_ = expected heterozygosity, F_IS_ = inbreeding coefficient, A_R_ = allelic richness, A_R_ (rarefied) = allelic richness estimated with rarefaction) for each population and each data type (microsatellites or putatively neutral SNPs). The “reduced” dataset is the microsatellite data for only the individuals included in the SNP data (N = population sample size).

|  |  |  | H_O_ | | H_E_ | | F_IS_ | | A_R_ | | A_R_ (rarefied) | |
| --- | --- | --- | --- | --- | --- | --- | --- | --- | --- | --- | --- | --- |
| Population | Data | N | Mean | SE | Mean | SE | Mean | SE | Mean | SE | Mean | SE |
| Cimarron | microsatellite | 4 | 0.534 | 0.067 | 0.413 | 0.046 | -0.279 | 0.109 | 2.183 | 0.155 | 2.455 | 0.194 |
| Crawford | microsatellite | 21 | 0.517 | 0.047 | 0.510 | 0.042 | -0.022 | 0.065 | 2.605 | 0.170 | 2.661 | 0.171 |
| Dove Creek | microsatellite | 43 | 0.464 | 0.036 | 0.514 | 0.043 | 0.071 | 0.049 | 2.602 | 0.165 | 2.656 | 0.166 |
| Gunnison Basin | microsatellite | 116 | 0.529 | 0.042 | 0.559 | 0.044 | 0.045 | 0.021 | 2.924 | 0.213 | 3.019 | 0.228 |
| Pinon Mesa | microsatellite | 19 | 0.498 | 0.041 | 0.505 | 0.036 | 0.007 | 0.047 | 2.428 | 0.128 | 2.539 | 0.139 |
| San Miguel | microsatellite | 51 | 0.548 | 0.034 | 0.578 | 0.037 | 0.038 | 0.022 | 2.853 | 0.171 | 2.923 | 0.177 |
| Cimarron | SNP | 4 | 0.183 | 0.002 | 0.154 | 0.002 | -0.194 | 0.005 | 1.354 | 0.004 | 1.410 | 0.004 |
| Crawford | SNP | 12 | 0.194 | 0.002 | 0.190 | 0.002 | -0.018 | 0.003 | 1.444 | 0.003 | 1.471 | 0.004 |
| Dove Creek | SNP | 12 | 0.189 | 0.002 | 0.189 | 0.002 | -0.004 | 0.003 | 1.440 | 0.004 | 1.470 | 0.004 |
| Gunnison Basin | SNP | 12 | 0.197 | 0.002 | 0.194 | 0.002 | -0.020 | 0.003 | 1.462 | 0.003 | 1.491 | 0.004 |
| Pinon Mesa | SNP | 10 | 0.185 | 0.002 | 0.177 | 0.002 | -0.041 | 0.004 | 1.412 | 0.004 | 1.439 | 0.004 |
| San Miguel | SNP | 10 | 0.196 | 0.002 | 0.193 | 0.002 | -0.019 | 0.004 | 1.448 | 0.004 | 1.480 | 0.004 |
| Cimarron | reduced | 4 | 0.534 | 0.070 | 0.413 | 0.048 | -0.279 | 0.073 | 2.178 | 0.155 | 2.455 | 0.194 |
| Crawford | reduced | 12 | 0.512 | 0.054 | 0.513 | 0.045 | 0.007 | 0.057 | 2.662 | 0.184 | 2.768 | 0.180 |
| Dove Creek | reduced | 12 | 0.462 | 0.049 | 0.498 | 0.046 | 0.063 | 0.048 | 2.519 | 0.173 | 2.622 | 0.182 |
| Gunnison Basin | reduced | 12 | 0.555 | 0.055 | 0.548 | 0.046 | 0.004 | 0.042 | 2.889 | 0.212 | 3.070 | 0.219 |
| Pinon Mesa | reduced | 10 | 0.513 | 0.051 | 0.491 | 0.041 | -0.041 | 0.055 | 2.386 | 0.141 | 2.549 | 0.154 |
| San Miguel | reduced | 10 | 0.549 | 0.046 | 0.539 | 0.042 | -0.030 | 0.035 | 2.622 | 0.167 | 2.824 | 0.200 |

Table S2. Differentiation statistics for Gunnison sage-grouse populations using different genetic marker types. Means and 95% CIs for all differentiation metrics (G_ST_, D_Jost_, F_ST_) for each population pair (Comparison) and each data type (microsatellites, putatively neutral SNPs, and all SNPs).

|  |  | G_ST_ | | D_Jost_ | | F_ST_ | |
| --- | --- | --- | --- | --- | --- | --- | --- |
| Comparison | Data | Mean | 95% CI | Mean | 95% CI | Mean | 95% CI |
| Cimarron-Crawford | microsatellite | 0.198 | [0.119 - 0.318] | 0.073 | [0.034 - 0.131] | 0.117 | [0.062 - 0.199] |
| Cimarron-Dove Creek | microsatellite | 0.323 | [0.240 - 0.450] | 0.153 | [0.115 - 0.210] | 0.181 | [0.131 - 0.256] |
| Cimarron-Gunnison Basin | microsatellite | 0.231 | [0.149 - 0.368] | 0.102 | [0.061 - 0.172] | 0.119 | [0.073 - 0.196] |
| Cimarron-Piñon Mesa | microsatellite | 0.528 | [0.450 - 0.639] | 0.284 | [0.213 - 0.384] | 0.292 | [0.241 - 0.367] |
| Cimarron-San Miguel | microsatellite | 0.300 | [0.229 - 0.399] | 0.110 | [0.077 - 0.163] | 0.148 | [0.106 - 0.207] |
| Crawford-Dove Creek | microsatellite | 0.313 | [0.262 - 0.361] | 0.175 | [0.138 - 0.211] | 0.179 | [0.147 - 0.209] |
| Crawford-Gunnison Basin | microsatellite | 0.160 | [0.129 - 0.195] | 0.086 | [0.069 - 0.109] | 0.088 | [0.070 - 0.109] |
| Crawford-Piñon Mesa | microsatellite | 0.543 | [0.495 - 0.599] | 0.339 | [0.289 - 0.400] | 0.291 | [0.259 - 0.329] |
| Crawford-San Miguel | microsatellite | 0.274 | [0.216 - 0.327] | 0.137 | [0.098 - 0.177] | 0.142 | [0.110 - 0.174] |
| Dove Creek-Gunnison Basin | microsatellite | 0.308 | [0.275 - 0.342] | 0.178 | [0.155 - 0.202] | 0.165 | [0.148 - 0.185] |
| Dove Creek-Piñon Mesa | microsatellite | 0.488 | [0.450 - 0.530] | 0.279 | [0.243 - 0.319] | 0.265 | [0.244 - 0.291] |
| Dove Creek-San Miguel | microsatellite | 0.272 | [0.227 - 0.317] | 0.151 | [0.120 - 0.180] | 0.145 | [0.119 - 0.169] |
| Gunnison Basin-Piñon Mesa | microsatellite | 0.478 | [0.447 - 0.513] | 0.272 | [0.238 - 0.309] | 0.243 | [0.227 - 0.263] |
| Gunnison Basin-San Miguel | microsatellite | 0.284 | [0.252 - 0.315] | 0.147 | [0.124 - 0.170] | 0.144 | [0.127 - 0.161] |
| Piñon Mesa-San Miguel | microsatellite | 0.459 | [0.422 - 0.497] | 0.250 | [0.215 - 0.288] | 0.228 | [0.206 - 0.251] |
| Cimarron-Crawford | neutral SNPs | 0.071 | [0.034 - 0.130] | 0.005 | [0.003 - 0.009] | 0.088 | [0.041 - 0.156] |
| Cimarron-Dove Creek | neutral SNPs | 0.186 | [0.153 - 0.235] | 0.016 | [0.013 - 0.021] | 0.218 | [0.181 - 0.270] |
| Cimarron-Gunnison Basin | neutral SNPs | 0.114 | [0.079 - 0.167] | 0.008 | [0.006 - 0.013] | 0.137 | [0.096 - 0.194] |
| Cimarron-Piñon Mesa | neutral SNPs | 0.259 | [0.223 - 0.311] | 0.025 | [0.022 - 0.031] | 0.303 | [0.263 - 0.356] |
| Cimarron-San Miguel | neutral SNPs | 0.140 | [0.097 - 0.198] | 0.011 | [0.008 - 0.016] | 0.167 | [0.115 - 0.232] |
| Crawford-Dove Creek | neutral SNPs | 0.146 | [0.120 - 0.175] | 0.014 | [0.012 - 0.017] | 0.178 | [0.146 - 0.214] |
| Crawford-Gunnison Basin | neutral SNPs | 0.071 | [0.053 - 0.092] | 0.006 | [0.005 - 0.008] | 0.090 | [0.067 - 0.118] |
| Crawford-Piñon Mesa | neutral SNPs | 0.226 | [0.202 - 0.256] | 0.024 | [0.022 - 0.028] | 0.266 | [0.236 - 0.303] |
| Crawford-San Miguel | neutral SNPs | 0.101 | [0.059 - 0.144] | 0.009 | [0.005 - 0.013] | 0.125 | [0.073 - 0.179] |
| Dove Creek-Gunnison Basin | neutral SNPs | 0.144 | [0.130 - 0.165] | 0.014 | [0.013 - 0.016] | 0.175 | [0.157 - 0.201] |
| Dove Creek-Piñon Mesa | neutral SNPs | 0.234 | [0.216 - 0.257] | 0.026 | [0.023 - 0.029] | 0.273 | [0.252 - 0.301] |
| Dove Creek-San Miguel | neutral SNPs | 0.120 | [0.101 - 0.147] | 0.011 | [0.010 - 0.014] | 0.147 | [0.123 - 0.180] |
| Gunnison Basin-Piñon Mesa | neutral SNPs | 0.233 | [0.217 - 0.256] | 0.025 | [0.023 - 0.028] | 0.272 | [0.252 - 0.299] |
| Gunnison Basin-San Miguel | neutral SNPs | 0.111 | [0.091 - 0.139] | 0.010 | [0.008 - 0.013] | 0.137 | [0.112 - 0.170] |
| Piñon Mesa-San Miguel | neutral SNPs | 0.215 | [0.193 - 0.243] | 0.023 | [0.020 - 0.026] | 0.253 | [0.227 - 0.289] |
| Cimarron-Crawford | all SNPs | 0.078 | [0.040 - 0.141] | 0.005 | [0.003 - 0.010] | 0.096 | [0.048 - 0.171] |
| Cimarron-Dove Creek | all SNPs | 0.210 | [0.178 - 0.262] | 0.019 | [0.016 - 0.025] | 0.242 | [0.206 - 0.296] |
| Cimarron-Gunnison Basin | all SNPs | 0.135 | [0.100 - 0.195] | 0.010 | [0.007 - 0.016] | 0.161 | [0.122 - 0.226] |
| Cimarron-Piñon Mesa | all SNPs | 0.285 | [0.252 - 0.342] | 0.029 | [0.025 - 0.036] | 0.328 | [0.292 - 0.389] |
| Cimarron-San Miguel | all SNPs | 0.157 | [0.114 - 0.223] | 0.013 | [0.009 - 0.019] | 0.185 | [0.135 - 0.258] |
| Crawford-Dove Creek | all SNPs | 0.165 | [0.138 - 0.193] | 0.016 | [0.013 - 0.019] | 0.197 | [0.164 - 0.232] |
| Crawford-Gunnison Basin | all SNPs | 0.086 | [0.068 - 0.109] | 0.007 | [0.006 - 0.009] | 0.107 | [0.084 - 0.138] |
| Crawford-Piñon Mesa | all SNPs | 0.248 | [0.222 - 0.277] | 0.027 | [0.024 - 0.031] | 0.286 | [0.256 - 0.323] |
| Crawford-San Miguel | all SNPs | 0.110 | [0.069 - 0.156] | 0.010 | [0.006 - 0.014] | 0.135 | [0.085 - 0.192] |
| Dove Creek-Gunnison Basin | all SNPs | 0.166 | [0.152 - 0.187] | 0.016 | [0.015 - 0.019] | 0.198 | [0.181 - 0.223] |
| Dove Creek-Piñon Mesa | all SNPs | 0.264 | [0.247 - 0.285] | 0.030 | [0.028 - 0.033] | 0.301 | [0.282 - 0.327] |
| Dove Creek-San Miguel | all SNPs | 0.139 | [0.119 - 0.168] | 0.013 | [0.011 - 0.017] | 0.168 | [0.143 - 0.204] |
| Gunnison Basin-Piñon Mesa | all SNPs | 0.258 | [0.241 - 0.281] | 0.028 | [0.026 - 0.031] | 0.296 | [0.277 - 0.324] |
| Gunnison Basin-San Miguel | all SNPs | 0.131 | [0.110 - 0.165] | 0.012 | [0.010 - 0.016] | 0.159 | [0.133 - 0.199] |
| Pinon Mesa-San Miguel | all SNPs | 0.236 | [0.214 - 0.265] | 0.026 | [0.023 - 0.029] | 0.274 | [0.249 - 0.309] |


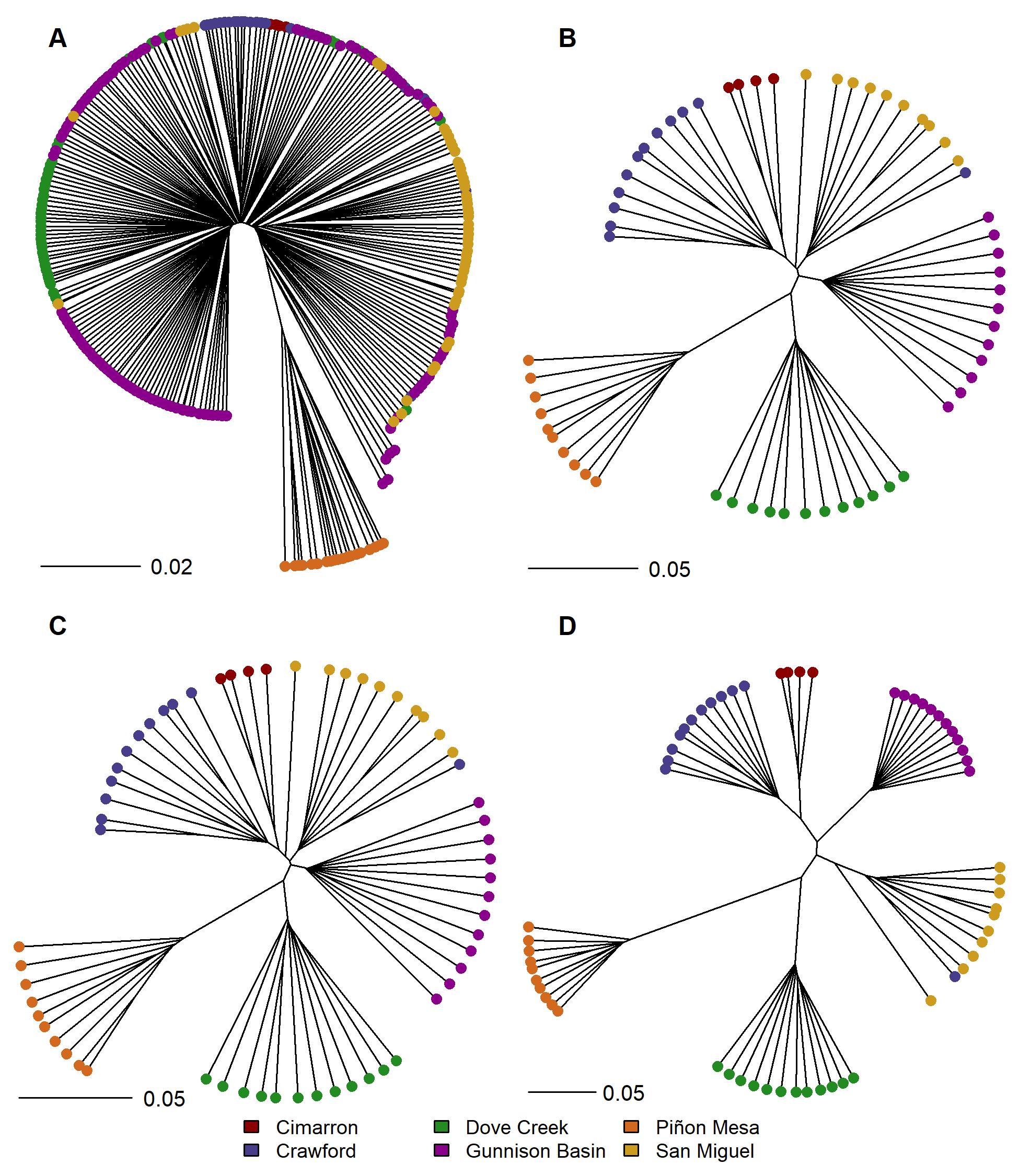


Figure S1. Dendrograms for Gunnison sage-grouse populations using different genetic marker types created using the single (based on closest pair) method. Each panel corresponds to a different data set: (A) microsatellites, (B) all SNPs, (C) putatively neutral SNPs, and (D) candidate adaptive loci.


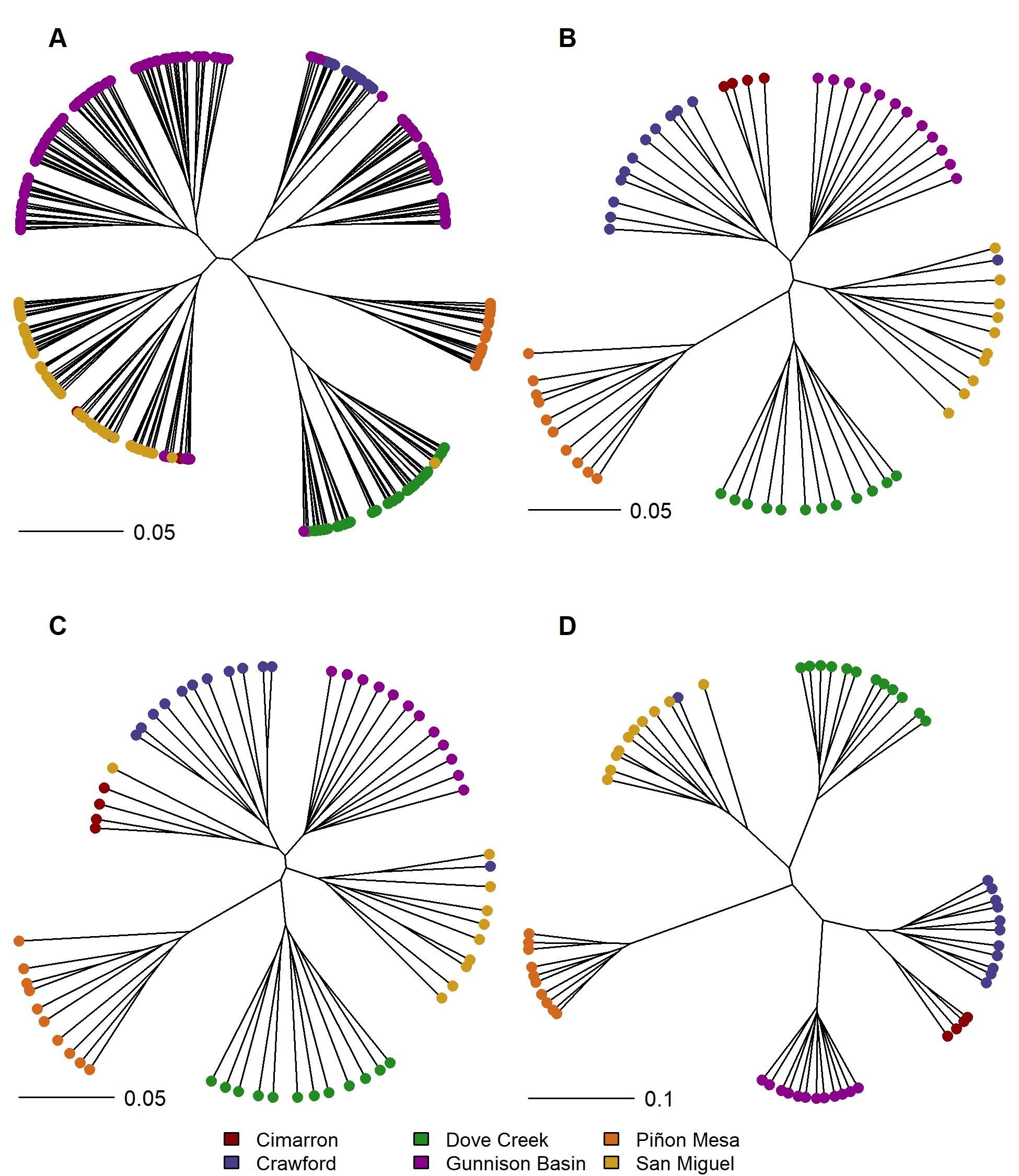


Figure S2. Dendrograms for Gunnison sage-grouse populations using different genetic marker types created using the complete (based on furthest pair) method. Each panel corresponds to a different data set: (A) microsatellites, (B) all SNPs, (C) putatively neutral SNPs, and (D) candidate adaptive loci.


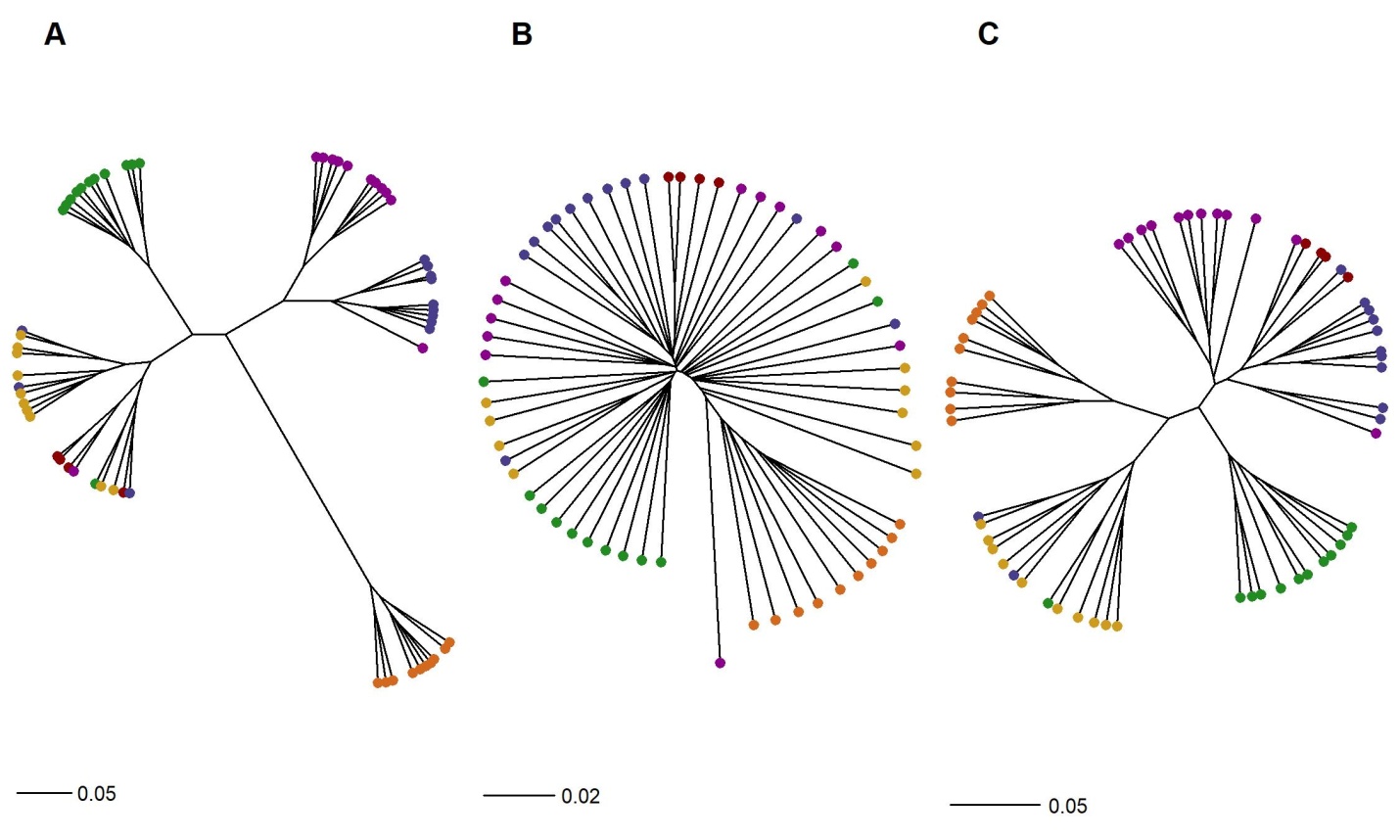


Figure S3. Dendrograms created using microsatellite loci from the 60 Gunnison sage-grouse individuals included in the SNP data set. Each panel corresponds to a different clustering algorithm: (A) “ward.D2” method, (B) “single” (based on closest pair), and (C) “complete” (based on furthest pair) method.


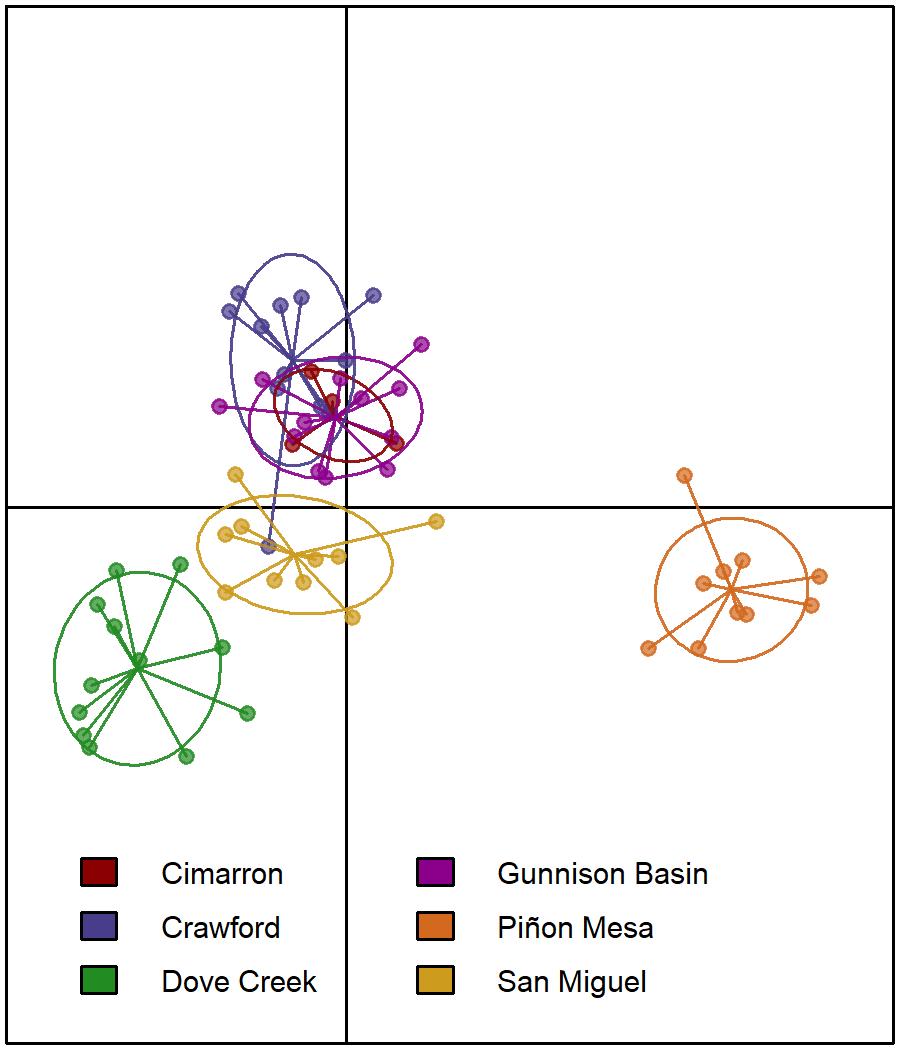


Figure S4. Star plot for the separation of Gunnison sage-grouse populations in a discriminant analysis of principle components (DAPC) for microsatellite from the 60 individuals sampled for SNPs: x-axis = discriminant function 1, y-axis = discriminant function 2.
